# Supplementary material for: Effects of a Lifestyle Intervention in Young Women with GDM and Subsequent Diabetes
Source: Nutrients. 2022 Dec 8;14(24):5232. doi: 10.3390/nu14245232 (PMC9785424; doi:10.3390/nu14245232)
Supplement: Supplementary file 1 [file nutrients-14-05232-s001.zip › nutrients-2073097-supplementary.pdf]

Table S1. Changes in body weight, energy intake and leisure time physical activity from baseline to the end of Months 1, 3, 6 and 9 visits among participants who finished follow-up visits

	Month 1	Month 3	Month 6	Month 9
No. of participants	61	63	63	68
Change in weight				
Kg	-1.78±2.10	-2.83±2.75	-3.18±3.26	-2.76±3.81
Percent reduction in initial weight	-2.50±3.00	-4.02±3.96	-4.52±4.66	-3.87±5.45
Change in energy intakes (kcal/day) [a]	-273±394	-277±373	-269±384	-58.9±362
Change in leisure time physical activity (minutes/day)	4.23±13.8	5.20±10.6	8.29±17.9	9.15±22.7

Data are changes in mean ± SD unless otherwise indicated.

[a] Dietary intakes are assessed by 3-day 24-hour food records.

Table S2. Changes in selected clinical and metabolic variables from baseline to the end of Month 9 among participants who finished follow-up visit

	Baseline	Month 9	Changes	P values
No. of participants	68	68	68	
Age at baseline or during follow-up (years)	33.2±3.90	34.3±3.95	1.16±0.45	
Body weight (kg)	69.3±11.7	66.5±11.6	-2.76±3.81	<0.001
Percent change in initial weight			-3.87±5.45	
Body mass index (kg/m^2)	27.1±4.23	26.0±4.25	-1.07±1.49	<0.001
Waist circumference (cm)	88.0±10.2	85.1±10.4	-2.88±6.00	<0.001
Body fat (%)	37.5±5.46	35.8±5.89	-1.69±2.31	<0.001
Systolic blood pressure (mmHg)	115±16.1	117±15.3	1.66±13.1	0.298
Diastolic blood pressure (mmHg)	81.2±11.8	77.7±11.3	-3.49±10.1	0.006
Fasting glucose (mmol/L)	7.73±2.27	6.75±2.06	-0.98±1.84	<0.001
Fasting insulin (pmol/L)	82.9±72.9	68.0±50.3	-14.9±60.5	0.047
HbA1c (%)	6.94±1.29	6.23±1.25	-0.71±1.04	<0.001
Total cholesterol (mmol/L)	4.91±0.91	4.87±0.96	-0.04±0.70	0.640
High-density lipoprotein cholesterol (mmol/L)	1.28±0.25	1.26±0.29	-0.02±0.24	0.468
Low-density lipoprotein cholesterol (mmol/L)	2.85±0.79	2.84±0.93	-0.01±0.77	0.956
Triglycerides (mmol/L)	1.69±0.78	1.61±1.75	-0.08±1.66	0.707
Use of glucose-lowering agents (%)	2.9	35.3	32.4	

Data are mean ± SD unless otherwise indicated.

Table S3. Changes in selected clinical and metabolic variables from baseline to Month 9 and Years 6-9

	Baseline	Month 9	Changes from baseline to Month 9	P values	Years 6-9	Changes from baseline to Years 6-9	P values
No. of participants	39	39	39		39	39	
Age at baseline or during follow-up (years)	33.7±3.83	34.8±3.92			40.5±3.98		
Follow-up from baseline (years)		1.12±0.34			6.79±1.13		
Body weight (kg)	70.0±10.2	67.3±9.48	-2.69±3.70	<0.001	66.5±8.88	-3.49±5.14	<0.001
Percent change in initial weight			-3.60±5.29			-4.56±7.09	
Body mass index (kg/m^2)	27.1±3.74	26.1±3.58	-1.02±1.43	<0.001	25.8±3.16	-1.35±2.04	<0.001
Waist circumference (cm)	88.3±8.90	85.6±9.04	-2.78±6.25	0.008	84.3±8.79	-4.03±7.05	0.001
Body fat (%)	37.7±4.84	36.2±4.97	-1.49±2.29	<0.001	35.8±4.56	-1.93±2.86	<0.001
Systolic blood pressure (mmHg)	115±12.6	115±13.2	-0.13±12.6	0.950	119±17.2	3.95±13.4	0.073
Diastolic blood pressure (mmHg)	81.6±9.96	77.5±9.84	-4.14±11.2	0.027	79.8±11.1	-1.81±7.74	0.153
Fasting glucose (mmol/L)	7.58±1.85	6.56±1.58	-1.02±1.77	0.001	8.24±2.80	0.66±2.77	0.143
Fasting insulin (pmol/L)	88.2±86.8	69.0±43.2	-19.2±66.6	0.078	75.7±48.8	-12.5±58.9	0.191
HbA1c (%)	6.83±1.16	6.14±0.97	-0.69±0.94	<0.001	7.09±1.55	0.26±1.67	0.339
Total cholesterol (mmol/L)	4.74±0.90	4.76±0.98	0.02±0.67	0.807	5.03±1.33	0.29±1.02	0.078
High-density lipoprotein cholesterol (mmol/L)	1.28±0.25	1.24±0.32	-0.04±0.23	0.306	1.32±0.30	0.04±0.23	0.278
Low-density lipoprotein cholesterol (mmol/L)	2.73±0.73	2.67±0.97	-0.06±0.80	0.638	2.91±0.87	0.18±0.71	0.131
Triglycerides, mmol/L	1.60±0.78	1.79±2.26	0.19±2.06	0.568	1.77±2.30	0.17±1.95	0.582
Use of glucose-lowering agents (%)	2.60	41.0	38.4		48.7	46.1	

Data are mean ± SD unless otherwise indicated.
